# Comparison of two main orthokeratology lens designs in effectiveness and safety for myopia control in different ages

**DOI:** 10.3389/fmed.2025.1681557

**Published:** 2025-10-28

**Authors:** Daoyuan Li, Qu Yang, Mengting He, Yinmao Yang, Min Liu, Shangkun Ou

**Affiliations:** ^1^Department of Ophthalmology, the Affiliated Hospital of Guizhou Medical University, Guiyang, Guizhou, China; ^2^Guizhou Medical University, Guiyang, Guizhou, China; ^3^Guizhou Nursing Vocational College, Guiyang, Guizhou, China; ^4^Guizhou Bright Eye Hospital, Guiyang, Guizhou, China

**Keywords:** orthokeratology, myopia control, CRT lenses, VST lenses, axial length, corneal curvature

## Abstract

**Purpose:**

Myopia represents the most prevalent ocular condition among children and adolescents worldwide, exhibiting marked variations in prevalence across regions and ethnic groups. This study aimed to assess and compare the effectiveness and safety of two orthokeratology (OK) lens types—corneal refractive therapy (CRT) and vision shaping treatment (VST)—for controlling myopia progression across different age groups.

**Methods:**

A retrospective cohort analysis was performed on 105 pediatric patients (210 eyes; aged 8–16 years) clinically diagnosed with myopia who wore CRT or VST lenses for at least 12 months. Longitudinal evaluations included axial length (AL) progression, axial length-to-corneal curvature (AL/CR) ratio, corneal curvature, corneal eccentricity indices (e-values), and safety parameters.

**Results:**

CRT lenses markedly limited AL elongation and reduced corneal curvature flattening in participants younger than 13 years (*p* < 0.0001), whereas VST lenses produced more favorable changes in E-values among those older than 11 years (*p* < 0.05). No significant difference was observed between the groups in the AL/CR ratio control (*p >* 0.05). Both lens designs maintained similar safety outcomes, with only mild corneal epithelial staining detected across all groups.

**Conclusion:**

The results reveal age-dependent variations in effectiveness, supporting an individualized approach to OK lens selection for optimized myopia management.

## 1 Introduction

Myopia represents the most prevalent ocular disorder among children and adolescents worldwide. Epidemiological studies have identified pronounced geographic and ethnic disparities, with East Asian populations exhibiting more than double the prevalence compared to age-matched Caucasian cohorts (1). Furthermore, incidence rates have continued to increase globally, particularly among younger age groups in East Asia (2). Over recent decades, this escalating trend has transformed myopia into a global public health concern, reaching pandemic proportions (3–5). Current estimates predict that by 2050, approximately 4.8 billion individuals—approximately half of the world’s population—will be affected by myopia. Given that uncorrected myopia remains a major cause of visual impairment, this condition constitutes a pressing public health issue requiring immediate attention (6, 7). Myopia is a complex refractive disorder marked by excessive axial length (AL) elongation and increased corneal power. Extensive evidence implicates both genetic susceptibility and environmental influences in its development (8). Progressive high myopia substantially heightens the risk of severe ocular complications, including open-angle glaucoma, cataracts, myopic macular degeneration, rhegmatogenous retinal detachment, and myopic choroidal neovascularization, often leading to irreversible vision loss later in life (9). Beyond ocular pathology, myopia negatively impacts children’s overall wellbeing, diminishing the quality of life through measurable effects on educational achievement, physical activity, social engagement, and future occupational opportunities (10).

Recognition of the extensive disease burden and pathological outcomes associated with myopia has prompted the intensive exploration of interventions aimed at mitigating its progression (11, 12). The current approaches include optical correction through spectacles, contact lenses, and surgical procedures. Despite substantial research, the precise mechanisms initiating and sustaining myopia remain only partially elucidated, leading to diverse theoretical interpretations. Among them, the peripheral retinal optical defocus hypothesis has gained substantial empirical support (13, 14). Peripheral retinal hyperopic defocus, marked by a posterior shift of the focal plane relative to the retina, induces choroidal thinning and promotes axial elongation, whereas peripheral myopic defocus (an anterior shift) results in choroidal thickening and slower axial elongation (15). This conceptual framework has informed the development of defocus-based clinical interventions, such as peripheral defocus spectacles (16, 17), defocus-incorporated soft contact lenses (18), and orthokeratology (OK) lenses (19, 20). The OK therapy has been validated as an effective modality for controlling pediatric myopia, with multiple studies confirming its capacity to slow axial elongation (11, 12, 21, 22). However, sustained therapeutic effectiveness generally necessitates consistent, long-term lens wear (23, 24). The expanding range of commercial OK lens designs introduces complexity in selecting the most suitable option for individualized treatment. Determining whether variations in lens design—particularly optical zone parameters—affect clinical performance is essential for optimizing therapeutic effectiveness. Increasing evidence indicates that lenses with smaller central treatment zones, as observed on corneal tangential maps, may exert stronger inhibitory effects on axial elongation (25–28). Reductions in optical zone diameter have been linked to the improved control of myopia progression, although comparative evaluations across lens designs remain limited (28). Despite these gaps, substantial data confirm the safety of OK as a corrective and therapeutic approach for myopia (23, 29–32). Nevertheless, continued investigation into lens design optimization is required to maximize treatment effectiveness while preserving ocular health.

The expanding scope of myopia-control strategies has prompted the emergence of varied orthokeratology lens configurations. Two principal systems prevail: corneal refractive therapy (CRT) and vision shaping treatment (VST). The CRT lens incorporates a tri-zone independent structure defined by specific parameters—base curve (BC), return zone depth (RZD), and landing zone angle (LZA). This structure supports sagittal height-based fitting, in which each zone can be precisely adjusted through modifications in sagittal height. Such a design enables controlled modulation of lens-induced mechanical forces via localized height alterations, with the LZA characterized by a tangential landing configuration. In contrast, VST lenses adopt a four-zone design integrating continuous transitional arcs comprising the BC, the return curve (RC), the alignment curve (AC), and the peripheral curve (PC). Optimization of fit is achieved by refining curvature radii across these interconnected segments (8). Clinical comparisons indicate that CRT lenses generally yield greater initial comfort and adaptability relative to VST lenses (33). The choice between designs depends on a combination of factors, including corneal topography conformity, parental preferences, and practitioner expertise. While earlier research largely examined orthokeratology effectiveness in relation to other myopia-control modalities, more recent studies have begun to delineate distinctions in treatment outcomes between CRT and VST systems (34). Despite these advances, substantial uncertainties remain regarding age-related variations in therapeutic response. The present study addresses this limitation by evaluating the comparative effectiveness of CRT and VST lenses across pediatric age groups, with the objective of refining age-specific clinical strategies for myopia management. To deepen insight into myopia control and determine whether variations in effectiveness and safety exist between different OK lens designs across pediatric age groups (35), a systematic 1-year clinical dataset including refractive error, AL, and ocular surface parameters was analyzed. The investigation focused on comparing the therapeutic performance and ocular safety of two major OK lens designs, CRT and VST. By conducting a comprehensive longitudinal assessment of biometric indices and ocular surface conditions, the study aimed to clarify age-related differences in treatment outcomes and provide evidence-based recommendations for selecting optimal lens designs in pediatric myopia management.

## 2 Patients and methods

### 2.1 Patients

This retrospective analysis involved 105 pediatric patients (210 eyes) aged 8–16 years who were diagnosed with myopia and had been wearing OK lenses for more than 1 year at Guizhou Pure Eye Hospital between October 2022 and October 2023. Participants were assigned to two groups: 45 patients (90 eyes) fitted with CRT lenses and 60 patients (120 eyes) fitted with VST lenses. The study complied with the principles of the Declaration of Helsinki.

### 2.2 Inclusion criteria

The inclusion criteria were as follows: (1) patients aged 8–16 years; (2) those with a cycloplegic spherical equivalent (SE) of ≤ − 6.00 D; (3) best-corrected visual acuity of ≥1.0 (Snellen chart) with no ocular abnormalities other than refractive error; (4) horizontal phoria of ≤15 prism diopters (△) at 6 m; (5) no congenital or systemic conditions influencing visual development; (6) absence of myopia-related fundus lesions; (7) corneal endothelial cell density ≥3,000/mm^2^; (8) normal intraocular pressure (IOP); (9) normal tear film break-up time (TBUT) and the Schirmer test; and (10) full compliance with follow-up schedules and complete clinical data.

### 2.3 Exclusion criteria

The exclusion criteria were as follows: (1) the inability to complete ophthalmic examinations; (2) history of ocular surgery, amblyopia, or strabismus; (3) family history of keratoconus; (4) corneal endothelial cell density <3,000/mm^2^; (5) dry eye syndrome (TBUT <10 s, Schirmer <10 mm/5 min); (6) previous keratitis; and (7) poor systemic condition.

### 2.4 Ophthalmic examinations

Comprehensive evaluations were conducted at baseline and at 1 day, 1 week, 1 month, and 1 year following lens fitting. Uncorrected visual acuity (UCVA) was assessed using a standardized logarithmic visual acuity chart (Snellen notation).

Cycloplegia was induced through three consecutive administrations of tropicamide–phenylephrine eye drops at 10-min intervals. Automated refraction (KR-800 autorefractor, Topcon Corporation) provided measurements of the spherical equivalent (SE = sphere + ½ cylinder).

Corneal integrity and tear film stability were examined through slit-lamp biomicroscopy (Carl Zeiss Meditec AG, Germany) using fluorescein sodium strips (Tianjin Jingming New Technology Development Co., Ltd.) for TBUT analysis.

Fundus imaging was obtained through digital fundus photography (Carl Zeiss Meditec AG, Germany).

IOP was determined by non-contact tonometry (Tianjin Wesso).

Corneal endothelial cell morphology was assessed through non-contact specular microscopy (SP-1P, Topcon Corporation, Japan).

AL was defined as the distance from the tear film to the retinal surface and measured via partial coherence interferometry (IOLMaster 500, Carl Zeiss Meditec AG, Germany).

Corneal topography was analyzed using the ATLAS 500 system (Carl Zeiss Meditec AG, Germany) to determine anterior corneal curvature and eccentricity (e-value).

### 2.5 OK Lens specifications and fitting protocols

CRT lenses: Paragon CRT (Paragon Vision Sciences, United States).

VST lenses: DreamVision (Epicon Medical Co., Ltd., China).

All fittings were conducted by certified optometrists in accordance with standardized, evidence-based orthokeratology protocols:

1. Parameter determination: Lens parameters were individually designed using corneal topography, AL, and refractive error data to ensure precise corneal alignment and target refractive correction.
2. Informed consent: Prior to treatment initiation, patients or guardians received detailed counseling outlining potential risks, therapeutic benefits, and adherence obligations, followed by written consent.
3. Lens fabrication: Lenses were custom-manufactured from gas-permeable fluorosilicone acrylate materials (DK ≥ 100 × 10^−11^ cm^2^·mL O₂/sec·mL·mmHg), ensuring optimal oxygen permeability and structural stability.
4. Quality assurance: Post-fabrication verification confirmed dimensional precision within ±0.02 mm and assessed surface uniformity through interferometric microscopy.
5. Patient education: Structured instruction sessions provided comprehensive guidance on lens application and removal, enzymatic disinfection using proprietary hydrogen peroxide systems, and early identification of potential complications.

### 2.6 Corneal injury grading

The severity of fluorescein staining was evaluated using Chu and Xie’s grading system (36): Grade 0, no or minimal punctate staining; Grade I, scattered punctate staining; Grade II, dense punctate staining accompanied by mild discomfort; Grade III, localized epithelial defects associated with moderate irritation; and Grade IV, extensive epithelial defects with severe symptoms.

### 2.7 Statistical analysis

Statistical analyses were conducted using SPSS 26.0. The normality of data distribution was verified using independent t-tests. Continuous variables were presented as mean ± standard deviation (x ± s). Intergroup comparisons were assessed through independent samples chi-squared tests. A value of *P* of < 0.05 was considered statistically significant.

## 3 Results

### 3.1 Baseline characteristics

A total of 45 patients (90 eyes) were assigned to the CRT group, and 60 patients (120 eyes) were assigned to the VST group. They were further stratified into four age subgroups (≤10, 11–12, 13–14, and ≥15 years). Baseline demographic variables, AL, and corneal curvature metrics showed no statistically significant differences between the groups (*p* > 0.05) (Table 1), confirming baseline comparability for subsequent effectiveness evaluation.

**Table 1:** Baseline characteristics.

Age (Y)	Group	Number of eyes	Male, %	SER (D)	AL (mm)	*p*-value
≤10 Y	CRT	40	45	43.260 ± 1.090	24.560 ± 0.672	0.061 0.744
	VST	66	42	42.710 ± 1.624	24.510 ± 0.813	
11–12 Y	CRT	18	44	43.390 ± 1.393	24.820 ± 0.443	0.076
	VST	22	64	42.600 ± 1.336	24.800 ± 0.851	0.929
13–14 Y	CRT	14	51	43.540 ± 1.319	25.130 ± 0.578	0.258
	VST	24	50	43.100 ± 1.020	25.050 ± 0.598	0.690
≥15 Y	CRT	18	48	42.310 ± 1.151	25.730 ± 0.707	0.366
	VST	8	50	42.710 ± 0.603	25.560 ± 0.864	0.602

CRT, corneal refractive therapy; VST, vision shaping treatment; SER, spherical equivalent refraction; AL, axial length.

### 3.2 Comparative UCVA outcomes of CRT versus VST lenses across age strata

Both CRT and VST lenses significantly improved UCVA across all age categories relative to pre-fitting baselines. Stratified analyses indicated that the magnitude of UCVA improvement did not differ significantly between the two lens types within any age group (*p* > 0.05) (Table 2), reflecting equivalent visual performance outcomes across designs.

**Table 2:** Comparison of UCVA between two kinds of OK lenses with age.

Age (Y)	Group	Uncorrected visual acuity					X2	*P*
		Before	After 1 D	After 1 W	After 1 M	After 1 Y		
≤10 Y	CRT	0.264 ± 0.163	0.753 ± 0.222	0.950 ± 0.136	1.038 ± 0.095	1.155 ± 0.150	0.014	1
	VST	0.251 ± 0.152	0.864 ± 0.233	0.940 ± 0.216	0.985 ± 0.053	1.040 ± 0.086		
11–12 Y	CRT	0.194 ± 0.123	0.750 ± 0.264	1.033 ± 0.128	1.067 ± 0.119	1.122 ± 0.167	0.031	1
	VST	0.295 ± 0.204	0.748 ± 0.291	0.938 ± 0.153	0.971 ± 0.127	1.019 ± 0.060		
13–14 Y	CRT	0.279 ± 0.244	0.717 ± 0.340	0.985 ± 0.099	1.031 ± 0.075	1.062 ± 0.096	0.01	1
	VST	0.232 ± 0.123	0.800 ± 0.276	1.050 ± 0.090	1.058 ± 0.093	1.050 ± 0.088		
≥15 Y	CRT	0.150 ± 0.071	0.777 ± 0.182	0.941 ± 0.112	0.982 ± 0.053	1.071 ± 0.099	0.018	1
	VST	0.105 ± 0.038	0.600 ± 0.288	0.825 ± 0.358	0.975 ± 0.158	1.000 ± 0.151		

CRT, corneal refractive therapy; VST, vision shaping treatment.

### 3.3 Age-stratified AL control effectiveness of CRT versus VST lenses

**Figure 1:**
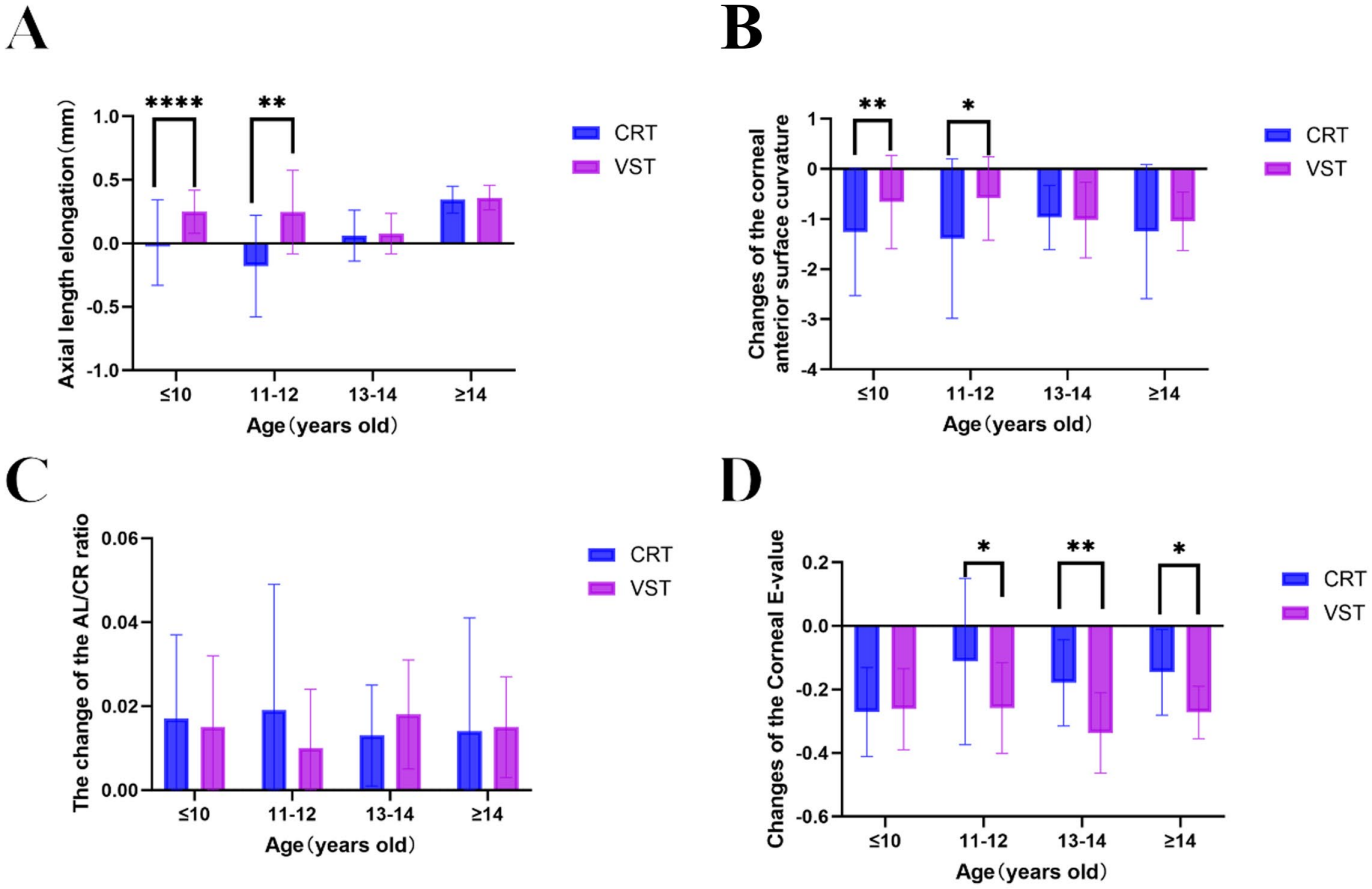
Comparative analysis of ocular parameter alterations following CRT and VST orthokeratology lens treatments over 1 year. **(A)** Age-dependent axial elongation. **(B)** Age-related variations in anterior corneal curvature (flat K). **(C)** Pre- and post-treatment changes in the axial length-to-corneal radius ratio (AL/CR). **(D)** Age-dependent modifications in corneal eccentricity (e-value). Data are expressed as mean ± SEM. *p < 0.05, **p < 0.01, ***p < 0.001, and ****p < 0.0001 indicate statistically significant intergroup differences at corresponding age intervals. CRT, corneal refractive therapy; VST, vision shaping treatment; SER, spherical equivalent refraction; AL, axial length; NS, not significant.

Both CRT and VST lenses effectively suppressed axial elongation over the 12-month follow-up. However, differences in age-related effectiveness were evident. Among participants younger than 13 years, AL progression was significantly slower in the CRT group (0.006 ± 0.337, −0.1786 ± 0.3999) than in the VST group (0.249 ± 0.171, 0.2462 ± 0.3293) (*p* < 0.05) (Supplementary Table 1; Figure 1A). The greatest difference occurred in the 11–12-year-old subgroup, where CRT lenses limited axial elongation by an additional 0.12 mm per year compared with VST lenses. In participants aged ≥13 years, both designs exhibited comparable effectiveness in axial growth control, with no statistically significant intergroup variation (*p* > 0.05) (Supplementary Table 1; Figure 1A).

### 3.4 Comparative effects of CRT and VST lenses on anterior corneal topography

Both CRT and VST lenses induced significant anterior corneal flattening across all age groups during the 1-year follow-up. Age-stratified comparisons, however, demonstrated marked design-specific variations in corneal remodeling effectiveness. In children younger than 13 years, CRT lenses produced substantially greater reductions in anterior corneal curvature than VST lenses (*p* < 0.05 for the 11–12-year cohort; *p* < 0.001 for the ≤10-year cohort) (Supplementary Table 2; Figure 1B). The degree of corneal flattening achieved with CRT exceeded that of VST by 0.42 ± 0.15 D in the ≤10-year-old subgroup and by 0.28 ± 0.11 D in the 11–12-year-old subgroup. Among adolescents aged ≥13 years, both lens designs induced comparable curvature modifications (*p* > 0.05), although CRT exhibited a non-significant inclination toward greater flattening. The age-related morphological responses closely mirrored the AL control outcomes, implying a potential mechanistic association between the efficiency of corneal remodeling and the suppression of myopia progression in younger individuals.

### 3.5 AL/CR in CRT versus VST groups

A comparative evaluation of the AL/CR ratio between baseline and the 1-year follow-up was conducted across age-stratified subgroups in both CRT and VST cohorts. No significant intergroup differences in AL/CR ratio changes were detected after orthokeratology treatment (*p* > 0.05) (Supplementary Table 3; Figure 1C). In the CRT group, post-treatment AL/CR ratios remained largely unchanged across all age subgroups, with minor deviations from baseline values (±0.03–0.05). The VST group exhibited a comparable pattern, maintaining similar stability in AL/CR ratios (±0.04–0.06), consistent with CRT outcomes. The results indicate that both lens designs induce proportionate alterations in AL and corneal curvature, thereby maintaining physiological AL/CR balance despite differences in lens geometry and corneal molding mechanics. The comparable AL/CR behavior across designs suggests similar biomechanical influences on ocular growth regulation, regardless of age-related variations in individual component responses. Although CRT achieved greater suppression of axial elongation and more pronounced corneal flattening in younger participants, the consistent AL/CR equilibrium observed in both designs implies that the overall ocular remodeling remains coordinated, likely reflecting intrinsic homeostatic adjustment within the visual system.

### 3.6 Alterations in e-value following CRT versus VST lens wear

Both CRT and VST lenses produced significant reductions in e-value across all age groups during the 1-year observation period. Age-stratified analysis, however, identified significant intergroup differences among participants older than 11 years. In the 11–12-year and ≥13-year cohorts, CRT lenses resulted in smaller decreases in corneal e-values compared with VST lenses (11–12 years: *p*< 0.05; ≥13 years: *p* < 0.001) (Supplementary Table 4; Figure 1D). Specifically, e-value reductions in the CRT group ranged from 0.08 ± 0.03 (11–12 years) to 0.12 ± 0.04 (≥13 years), whereas the VST group exhibited larger declines of 0.15 ± 0.05 (11–12 years) and 0.24 ± 0.06 (≥13 years). In contrast, participants aged ≤10 years showed comparable changes between designs (*p* > 0.05). These outcomes indicate that lens-induced corneal remodeling exhibits age-dependent biomechanical variability, with CRT lenses producing more moderate eccentricity alterations in older children.

### 3.7 Comparative corneal epithelial integrity in CRT versus VST groups

Fluorescein staining revealed mild epithelial alterations in both groups following lens wear. No significant intergroup differences were detected in the frequency or severity of corneal injury (*p* > 0.05) (Table 3), indicating comparable epithelial tolerance between. CRT and VST designs.

**Table 3:** Comparison of the incidence of total adverse events over a 1-year period of different OK lens wear.

Age (Y)	Group	Corneal fluorescein stain grading				X2	*P*
		0(%)	I (%)	II (%)	III/IV (%)		
≤10 Y	CRT	88.75	9.38	1.88	0	1.139	0.569
	VST	93.08	5.77	1.15	0		
11–12 Y	CRT	100	0	0	0	22.692	<0.0001
	VST	79.76	20.24	0	0		
13–14 Y	CRT	90.38	5.77	3.85	0	4.155	0.125
	VST	92.05	7.75	0	0		
≥15Y	CRT	88.71	11.29	0	0	0.199	0.655
	VST	90.63	9.37	0	0		

CRT, corneal refractive therapy; VST, vision shaping treatment.

## 4 Discussion

Our age-stratified analysis revealed a distinct pattern in orthokeratology effectiveness, demonstrating that CRT and VST lenses exhibit age-dependent therapeutic differences. Superior control of axial elongation and greater corneal flattening was achieved with CRT in children under 13 years, whereas greater modulation of corneal asphericity (e-value) by VST was observed in adolescents aged 11 years and above, indicating that patient age serves as a determinant factor in individualized lens selection.

The underlying mechanism for this divergence likely stems from the interaction between lens biomechanics and age-related ocular characteristics (37). The enhanced performance of CRT in younger children may result from higher corneal bioelasticity. The tri-zone structure of the CRT lens—incorporating a smaller central treatment zone and a steeper return zone—promotes substantial corneal reshaping in the more compliant juvenile cornea, producing pronounced central flattening and mid-peripheral steepening. Such remodeling mitigates peripheral hyperopic defocus, a primary stimulus for axial elongation in this vulnerable cohort (34, 38).

Conversely, the observed advantage of VST in modulating e-values among older adolescents may reflect biomechanical adaptation in a structurally stiffer cornea. With reduced corneal pliability after puberty, the VST configuration may induce a more balanced redistribution of corneal tension, enhancing optical performance through controlled asphericity adjustment rather than extensive flattening (39, 40). This normalization of eccentricity may improve retinal image quality and modulate visual signaling, contributing to myopia control through an alternative mechanistic route.

Notably, the consistent AL/CR ratio across all age groups and lens types suggests that both lens designs induce proportionate modifications in ocular dimensions, despite distinct mechanical mechanisms (41). This maintenance of overall ocular geometry reflects the integrative impact of OK therapy on eye structure.

The similar safety outcomes, characterized solely by transient and mild epithelial staining in both cohorts, further affirm the short-term tolerability of each design in children.

Several limitations warrant attention, including the 1-year observation period and the relatively moderate cohort size. Broader and longer-term investigations are needed to substantiate the persistence of age-dependent outcomes. Additionally, sequential or combined regimens—employing CRT during early adolescence to achieve stronger AL regulation, followed by or integrated with VST for refined optical performance—may represent a promising direction for future research.

In summary, the results highlight the importance of age-sensitive applications in orthokeratology. CRT appears more advantageous for younger patients requiring intensive control of axial elongation, whereas VST may confer benefits in older adolescents through enhanced management of corneal asphericity. Identifying this interaction between age and lens design constitutes an essential advancement toward precision-based myopia management.

## Data Availability

The original contributions presented in the study are included in the article/Supplementary material, further inquiries can be directed to the corresponding author.
