# Supplementary material for: Comparison of two main orthokeratology lens designs in effectiveness and safety for myopia control in different ages
Source: Front Med (Lausanne). 2025 Oct 28;12:1681557. doi: 10.3389/fmed.2025.1681557 (PMC12602486; doi:10.3389/fmed.2025.1681557)
Supplement: Supplementary file 1 [file Table_1.docx]

**Supplementary Table 1. The axial elongation with age after treatment with CRT and VST.**

| **Age（Y）** | **Axial length elongation（mm）** | | ***95%CI*** | ***P* value** |
| --- | --- | --- | --- | --- |
|  | **CRT** | **VST** |  |  |
| **≤10** | 0.006±0.337 | 0.249±0.171 | 0.1301-0.3552 | <0.0001^a^ |
| **11-12** | -0.1786±0.3999 | 0.2462±0.3293 | 0.1330-0.7164 | 0.0061 ^a^ |
| **13-14** | 0.06091±0.1993 | 0.07609±0.159 | -0.1137-0.1440 | 0.8119 |
| **≥15** | 0.3435±0.1053 | 0.36±0.09725 | -0.07480-0.1077 | 0.7124 |

CRT: corneal refractive therapy; VST: vision shaping treatment; CI: confidence interval; a:P<0.05

**Supplementary Table 2. The changes of the corneal anterior surface curvature with age after treatment.**

| **Age（Y）** | **Changes of the corneal anterior surface curvature (D)** | | ***P* value** |
| --- | --- | --- | --- |
|  | **CRT** | **VST** |  |
| **≤10** | -1.256±1.268 | -0.658±0.932 | <0.008^a^ |
| **11-12** | -0.1786±0.400 | 0.246±0.329 | 0.006 ^a^ |
| **13-14** | 0.061±0.199 | 0.0761±0.159 | 0.812 |
| **≥14** | 0.3440±0.105 | 0.360±0.0973 | 0.712 |

CRT: corneal refractive therapy; VST: vision shaping treatment, a:*P*<0.05

**Supplementary Table 3. The changes of the AL/CR ratio with age after treatment**

| **Age（Y）** | **Changes of the AL/CR ratio** | | ***P* value** |
| --- | --- | --- | --- |
|  | **CRT** | **VST** |  |
| **≤10** | 0.017±0.020 | 0.015±0.017 | 0.660 |
| **11-12** | 0.019±0.030 | 0.010±0.014 | 0.232 |
| **13-14** | 0.013±0.012 | 0.018±0.013 | 0.279 |
| **≥14** | 0.014±0.027 | 0.015±0.012 | 0.886 |

CRT: corneal refractive therapy; VST: vision shaping treatment; AL: axial length; CR: corneal anterior surface curvature; a:P<0.05.

| **Age（Y）** | **Changes of the corneal E-value** | | ***P* value** |
| --- | --- | --- | --- |
|  | **CRT** | **VST** |  |
| **≤10** | -0.271±0.140 | -0.262±0.127 | 0.724 |
| **11-12** | -0.112±0.262 | -0.259±0.143 | 0.036^a^ |
| **13-14** | -0.179±0.136 | -0.337±0.127 | 0.003 ^a^ |
| **≥14** | -0.146±0.135 | -0.273±0.083 | 0.027 ^a^ |

**Supplementary Table 4. The changes of the corneal E-value with age after treatment**

CRT: corneal refractive therapy; VST: vision treatment; a:*P*<0.05
